# Concentrations of Selected Adipokines, Interleukin-6, and Vitamin D in Patients with Papillary Thyroid Carcinoma in Respect to Thyroid Cancer Stages

**DOI:** 10.1155/2018/4921803

**Published:** 2018-12-03

**Authors:** Jakub Warakomski, Ewa Romuk, Barbara Jarząb, Jolanta Krajewska, Lucyna Siemińska

**Affiliations:** ^1^Department of Pathophysiology and Endocrinology, School of Medicine with the Division of Dentistry, Medical University of Silesia, Zabrze, Poland; ^2^Department of Biochemistry, School of Medicine with the Division of Dentistry, Medical University of Silesia, Zabrze, Poland; ^3^Nuclear Medicine and Endocrine Oncology Department, Maria Sklodowska-Curie Memorial Cancer Centre and Institute of Oncology, Gliwice Branch, Gliwice, Poland

## Abstract

The relationships between thyroid cancer and obesity are not fully understood. Adipokines, proinflammatory cytokines, and vitamin D may mediate these associations. In this study, we estimated serum concentrations of leptin, adiponectin, chemerin, interleukin-6 (IL-6), and vitamin D in patients with papillary thyroid cancer (PTC). We searched for associations between the adipokines, IL-6, vitamin D, anthropometric parameters, and TNM AJCC/UICC 2017 classification in 177 patients diagnosed with PTC (151 women and 26 men). Normal weight patients were predominantly classified as clinical stage I. The prevalence of stages higher than I was significantly higher in PTC patients with BMI ≥ 25 or with metabolic syndrome. Using logistic regression waist circumference ≥ 88 cm in women and ≥102 cm in men, upper tertiles of IL-6 and leptin were associated with a higher clinical stage. There were no differences in the prevalence of microcarcinomas in analyzed groups (BMI ≥ 25 versus BMI < 25 and with metabolic syndrome presence versus without metabolic syndrome). No significant relationships between serum concentrations of leptin, adiponectin, chemerin, IL-6, vitamin D, and tumor size in PTC were found. Although insulin resistance represented by the HOMA index was associated with anthropometric variables and with serum leptin, adiponectin, chemerin, and IL-6 concentrations, in our study, no statistically significant relations with PTC staging were identified.

## 1. Introduction

Thyroid carcinoma is the most common endocrine malignant neoplasm [1], and its incidence has been progressively increasing over the last years [2], but the explanation of this phenomenon is unknown. The highest incidence rate is noted in the 4th and 5th decades of life. Women are affected three times more frequently than men. About 1500-1800 new cases are diagnosed annually in Poland. Thyroid carcinomas arise from papillary thyroid cells and include differentiated cancers (90%) and nondifferentiated (anaplastic) thyroid cancer (5%). Medullary tumors originate from the parafollicular cells (C cells) of the thyroid gland. Differentiated thyroid carcinoma (DTC) is the most prevalent thyroid cancer, including papillary thyroid cancer (PTC) and follicular thyroid cancers (FTC). Fine-needle aspiration biopsy and histopathological evaluation are necessary for a definitive diagnosis.

The prevalence of DTC has increased in recent years. The aetiology is related to environmental and genetic factors, iodine intake, and ionising radiation [3]. Over the last decade, researchers have discussed the correlations between the adipose tissue, BMI, insulin resistance, and DTC [4, 5]. Diabetes and insulin resistance are associated with the increased risk of DTC [6] whereas metformin is associated with the inhibition of cancer cell growth [7].

The adipose tissue secretes a number of adipokines, which may promote cancerogenesis and increase tumor progression. A negative correlation was found between adiponectin concentration and the risk of breast, endometrial, and colorectal cancers [8]. However, the relationships with DTC are not fully understood. The overexpression of leptin and its receptors has been described in numerous types of cancer [9]. Currently, the role of leptin in the pathogenesis of DTC is also reported [10, 11]. Chemerin, another adipokine, stimulates chemotaxis of dendritic cells, macrophages, and NK cells to inflammatory sites. On the other hand, it shows anti-inflammatory properties by reducing secretion of proinflammatory cytokines (TNF-*α* and IL-6) and by stimulating adiponectin production [12]. To the best of our knowledge, there have been no studies on chemerin concentrations in patients with DTC. The role of proinflammatory cytokines and vitamin D in DTC still remains unaccounted for.

It was demonstrated that colorectal, pancreatic, liver, endometrial, and breast cancers are more prevalent in patients with the metabolic syndrome. However, the correlation between this syndrome and DTC is still not fully understood. Some researchers report that in patients with the metabolic syndrome, PTC occurs more frequently as compared to the general population [13], and the risk of thyroid cancer increases with rising BMI and serum glucose level [14]. Elevated TSH levels are found in patients with the metabolic syndrome, and this hormone is known to be a growth factor for DTC [15].

The aims of the study were to determine serum concentrations of selected adipokines (i.e., leptin, adiponectin, and chemerin), IL-6, and vitamin D in patients with a predominant histological type of thyroid malignancy—PTC, and to assess the associations between obesity, metabolic syndrome, examined adipokines, and tumor staging.

## 2. Materials and Methods

We analyzed data of 177 patients (151 women and 26 men) with newly diagnosed PTC, who were operated on after PTC diagnosis by fine-needle aspiration biopsy, whose final diagnosis of malignancy was proven on histology findings. The subjects were recruited from patients who were referred for treatment to the Department of Nuclear Medicine and Endocrine Oncology, Maria Sklodowska-Curie Memorial Cancer Centre and Institute of Oncology, Gliwice Branch, Poland. The TNM AJCC/UICC 2017 staging system (8th edition) was used for cancer staging. Data of patients and blood samples were collected preoperatively. The exclusion criteria were as follows: the presence of other cancer diseases, kidney or hepatic failure, and diabetes.

Written informed consent was obtained from all participants. The study was approved by the Bioethics Committee. Anthropometric measurements of height (cm), weight (kg), and waist and hip circumference (cm) were done after outpatient visits. Next, BMI and WHR were calculated. Blood pressure was measured at rest, and fasting venous blood samples were collected from each participant in the morning during routine outpatient visits. The blood samples were centrifuged, and the samples were stored at −70°C until assays were performed.

We assessed the concentrations of leptin, adiponectin, chemerin, interleukin-6 (IL-6), vitamin D (25OHD), total cholesterol, HDL, TGs, glucose, and insulin. HOMA-IR was calculated according to the following formula: fasting glucose (mg/dL/18.1) × fasting insulin (*μ*IU/mL/22.5). In the current study for diagnosis of insulin resistance, we used the threshold value HOMA-I ≥ 2.0.

Absence or presence of the metabolic syndrome was assessed according to the NCEP/ATP III (2001) criteria, modified by the AH/NHLBI criteria (2005). The following were considered risk factors: waist circumference ≥ 88 cm (women) or ≥102 cm (men), HDL concentration < 50 mg/dL (women) or <40 mg/dL (men), TG concentration > 150 mg/dL, glucose level ≥ 100 mg/dL, and BP > 130/85 mmHg. Diagnosis of the metabolic syndrome was established if at least 3 risk factors were present.

Biochemical analyses were performed using ELISA (commercial kits) or basic laboratory tests (in the case of glucose, total cholesterol, HDL, and TGs) at the Department of Pathophysiology and Endocrinology of the Medical University of Silesia. Intra- and extra-assay errors did not exceed 10%.

Patients with PTC were stratified into the following subgroups:

- With normal BMI (BMI < 25) versus with BMI higher than normal (BMI ≥ 25)
- With the metabolic syndrome versus without the metabolic syndrome

Patients with PTC were also divided according to the AJCC classification, 8th edition (TNM staging and AJCC prognostic clinical stage grouping). For the statistical analysis, we separated cases into groups based on tumor size (≤1 cm versus >1 cm).

Statistical analysis was performed with STAT SOFT STATISTICA v.13. The means, standard deviations (SD), and medians were calculated. The normality of the distribution was assessed using the Shapiro-Wilk test. Nonparametric tests were used when the distributions of the variables were not normal. Groups were compared by Student's test or Mann-Whitney *U* test. Correlations between variables were estimated by calculating the correlation coefficient *R* by Pearson's method or Spearman's method. The prevalence of categorical variables were compared using the chi-squared test. Logistic regression analysis was used to assess the association between the PTC clinical stage and different measures of adiposity (waist circumference and BMI) and serum concentrations of adipokines, interleukin-6, and vitamin D. To perform the analysis, continuous variables were divided into tertiles and we compared the upper and the lower tertiles. The odds ratios (ORs) and 95% confidence intervals (CIs) were estimated; all analyses were adjusted by sex. The level of *p* < 0.05 was considered statistically significant.

## 3. Results

One hundred and seventy-seven patients were enrolled in the study, i.e., 151 women (mean age 50.50; SD 14.38) and 26 men (mean age 53.84; SD 13.03). After TNM classification, 94 patients had pT1a, 37 pT1b, 14 pT2, 30 pT3, and 2 pT4. Nodal metastases were observed in 37 patients (10 in pT1a, 10 in pT1b, 4 in pT2, 11 in pT3, and 2 in pT4), while 140 patients had no metastases. Only one patient had distal metastases.

One hundred forty-four patients (125 women and 19 men) were qualified as having stage I, 31 patients (25 women and 6 men) had stage II, 1 woman had stage III, and 1 man had stage IVB. Detailed characteristics of the PTC patients are presented in Table 1.

The subjects were grouped according to BMI. Fifty-one women (33.77%) were of normal weight; 100 (66.23%) were overweight or obese. Twenty-six men (100%) had BMI ≥ 25. Clinical and biochemical data of patients with PTC depending on the BMI are shown in Table 2.

No statistically significant differences were found in microcarcinomas with the increase of BMI. However, clinical stages higher than I were more frequent in overweight/obese cases.

The metabolic syndrome was diagnosed in 68 patients.  Table 3 shows the comparison of anthropometric and biochemical parameters depending on the presence/absence of the metabolic syndrome.

Higher prevalence of stage II was observed in patients with metabolic syndrome. Microcarcinomas were equally likely to occur in both groups. Significantly higher concentrations of leptin, chemerin, IL-6, and HOMA-IR and decreased adiponectin concentrations were observed in patients with the metabolic syndrome.

In the next step of our study, we divided the PTC subjects into 2 tumor size categories.  Table 4 shows the characteristics of 94 patients with microcarcinoma (pT1a) and 83 patients with higher grades of cancer (>pT1a). In comparison with patients with tumor diameter ≤ 1 cm versus those with tumor diameter > 1 cm, there were no significant differences.

Clinical stage at time of diagnosis is used to determine the prognosis of disease; therefore, it is important to identify risk factors for staging. To investigate whether clinical stages higher than I are associated with obesity, insulin resistance, and serum concentrations of adipokines, interleukin-6, and vitamin D, we applied logistic regression analysis. Due to small sample size for stages III and IV, the cancer stage was dichotomized as stage I and stages higher than I (II, III, and IV). Continuous variables were categorized as low, medium, or high based on tertiles of distribution. Sex was included in the analysis as an additional factor.

As seen in Table 5, cases with BMI ≥ 25 tended to have a higher clinical stage. Odds ratio for subjects with BMI ≥ 25 was 5.0 (95% confidence interval (CI) 1.44-17.36). Patients with abdominal adiposity were also more likely to have a higher clinical stage. Odds ratio for those with waist circumferences ≥ 88 cm in women and ≥102 cm in men was 6.23 (95% CI 1.78-21.89). Furthermore, we observed increased risk of higher clinical stages in subjects with serum IL-6 concentrations in the upper tertile, compared with cases whose serum IL-6 were in the lower tertile (OR 8.05, 95% CI 2.19-29.56). Similar interactions were identified with upper/lower tertiles of leptin (OR 3.9, 95% CI 1.18-13.01). Patients classified as having stage I versus higher than stage I were similar with respect to adiponectin, chemerin, and vitamin D tertiles and HOMA-I. The sex of the patients seems not to affect the above conclusion.

In the last step of the study, we assessed the correlations between HOMA-I and analyzed variables in all subjects. We observed significant associations with BMI, waist circumference, WHR, TGs, HDL, leptin, adiponectin, chemerin, and IL-6 (Table 6).

In our study, 93 patients (52.54%) had insufficient vitamin D levels (<75 nmol/L). 25OHD concentrations showed negative correlations with BMI (*R* = −0.17, *p* < 0.05). However, no associations were found with tumor size or clinical stage.

## 4. Discussion

Epidemiological dates indicate that increased BMI is a risk factor for several cancers, including thyroid cancer [13, 14]. Some studies suggest that obesity is associated with larger tumor size and advanced TNM stage [15]. In our study conducted on 177 patients, we did not observe a relationship between BMI and tumor size; however, overweight/obese patients more often had a higher clinical stage of PTC. We found that especially central adiposity was connected with a higher prognostic stage. Our results are consistent with data presented by Schmid et al. The authors showed that a 0.1-unit increase in waist-to-hip ratio was associated with 14% higher risk of thyroid cancer [16]. Visceral fat is a strong predictor of insulin resistance, and some studies have confirmed the association between insulin resistance and PTC [5, 6]. In this study no significant statistical correlation between HOMA-I and clinical stage was observed; however, higher prevalence of a more advanced clinical stage was seen in patients with metabolic syndrome. Similarly, another studies reported that metabolic syndrome and its components increase thyroid cancer [13, 17]. In our study, we found that such variables such as BMI, waist circumference, WHR, TGs, HDL, leptin, adiponectin, chemerin, and IL-6 are related to HOMA-I, which in turn is related to metabolic syndrome. In addition, logistic regression showed that patients with higher preoperative serum concentrations of leptin and IL-6 were more likely to have a more advanced clinical stage, independent of sex. It is therefore likely that mechanisms linking abdominal obesity and thyroid cancerogenesis include adipokine and cytokine effects. It is well documented that leptin stimulates tumor cell growth and invasion. The significance of hyperleptinemia in breast, colorectal, and hepatocellular cancer has been well documented [18], and some studies suggest the role of leptin in thyroid cancerogenesis [11]. Higher serum levels of leptin in patients with DTC compared to those in the control group were observed by Akinci et al. [10] and Hedayati et al. [19]. Moreover, leptin and OBR expression levels in papillary thyroid cancer samples are associated with tumor size [11]. In our study, after dividing the PTC patients into two groups based on tumor size, we did not observe the differences in serum leptin concentrations. However, higher leptin levels were connected with a more advanced clinical stage of PTC.

Previous studies concerning relationship between adiponectin and thyroid cancer have reported inconclusive results [20]. Generally, adiponectin levels have been seen to be reduced in several cancers: breast, colorectal, endometrial, gastric, esophageal, pancreatic, hepatic, renal, prostate, and lung [8]. Most of researchers have proven that lower adiponectin is associated with more aggressive clinicopathological features and lower survival rates. However, some studies have reported contradictory results, suggesting that higher serum adiponectin was related to cancer progression and worse prognosis [21, 22]. In our study, patients with metabolic syndrome had lower serum adiponectin concentrations, but we did not find associations with tumor size or clinical stage of disease.

Chemerin, one of the newly discovered adipocytokines, shows a strong correlation with metabolic syndrome and obesity [12]. Plasma concentration of chemerin is closely associated with BMI, waist circumferences, and TG concentrations. In the last years, the potential role of chemerin in cancerogenesis was discussed [23, 24]. Higher chemerin levels in non-small cell lung cancer patients compared with healthy controls were observed, and this adipokine was associated with a more advanced TNM stage [24]. However, the results of our study do not confirm that chemerin directly contributes to the PTC.

Large epidemiological studies have shown that vitamin D deficiency raises the risk of several cancers. In our study, most of the patients had insufficient vitamin D levels and 25OHD concentrations showed negative correlations with BMI. However, no associations were detected between vitamin D and the clinical stage of the disease. The results are consistent with the study of Ahn et al. [25].

Numerous proinflammatory cytokines play a significant role in thyroid cancerogenesis. Infiltration of inflammatory and immune cells promotes development and growth of neoplastic thyroid cells [26]. The local process may be related to systemic inflammation characterized by high concentrations of cytokines. In our study, we found associations between IL-6 with clinical stages higher than I, and it confirms the previous reports [26, 27].

## 5. Conclusions

Normal weight PTC patients were predominantly classified as clinical stage I. The prevalence of clinical stages higher than I was significantly higher in the PTC patients with BMI ≥ 25 or with metabolic syndrome.

Using logistic regression waist circumference ≥ 88 cm in women and ≥102 cm in men, upper tertiles of IL-6 and leptin were associated with higher clinical stages.

There were no differences in the prevalence of microcarcinomas in the analyzed groups (BMI ≥ 25 versus BMI < 25 and with metabolic syndrome presence versus without metabolic syndrome).

No significant relationships between serum concentrations of leptin, adiponectin, chemerin, IL-6, vitamin D, and tumor size in PTC were found.

Although insulin resistance represented by the HOMA index was associated with anthropometric variables and with serum leptin, adiponectin, chemerin, and IL-6 concentrations, in our study, no statistically significant relations with PTC staging were identified.

## Figures and Tables

**Table 1:** Clinicopathological data of the study participants.

Characteristics	*N* (%)
Sex	
Women	151 (85.31)
Men	26 (14.69)
Age	
<55 years	86 (48.59)
≥55 years	91 (51.41)
Tumor size	
≤1 cm	94 (53.11)
>1 cm	83 (46.89)
Nodal status	
N0	140 (79.10)
N1a	29 (16.38)
N1b	8 (4.52)
Distant metastasis	
No	176 (99.43)
Yes	1 (0.56)
TNM stage	
I	144 (81.36)
II	31 (17.51)
III	1 (0.56)
IVA/IVB	0/1 (0.56)

**Table 2:** Anthropometric and biochemical characteristics and clinical stage of patients with PTC depending on the BMI.

	BMI < 25 *n* = 51 51 women, 0 men (mean ± SD; median)	BMI ≥ 25 *n* = 126 100 women, 26 men (mean ± SD; median)	*p*
Age	41.16 ± 11.86; 38	55.38 ± 12.68; 58	<0.001
BMI (kg/m^2^)	21.75 ± 2.08; 21.45	30.65 ± 4.05; 30.11	<0.001
Waist (cm)	75.73 ± 8.29; 75	99.28 ± 11.21; 98	<0.001
WHR	0.78 ± 0.07; 0.78	0.90 ± 0.08; 0.89	<0.001
Glucose (mg/dL)	122.91 ± 24.72; 117	119.84 ± 25.77; 115	NS
TGs (mg/dL)	116.05 ± 48.35; 110	175.36 ± 69.99; 170	<0.001
HDL (mg/dL)	59.17 ± 18.60; 52	50.01 ± 18.65; 53	NS
Cholesterol (mg/dL)	249.41 ± 106.37; 212	287.47 ± 125.00; 232	NS
HOMA-IR	1.11 ± 0.67; 0.92	1.44 ± 1.03; 1.21	0.06
Adiponectin (*μ*g/mL)	15.82 ± 6.67; 15.03	14.60 ± 6.23; 13.42	NS
Leptin (ng/mL)	8.54 ± 5.73; 7.18	22.25 ± 19.14; 18.84	<0.001
Chemerin [ng/mL]	211.74 ± 47.21; 214.50	229.27 ± 50.11; 223.00	<0.05
Vitamin D (nmol/L)	68.00 ± 23.99; 63.00	65.08 ± 22.84; 61.00	NS
IL-6 (pg/mL)	2.19 ± 0.80; 2.00	2.87 ± 1.01; 2.61	<0.001
*N* (%) of patients with microcarcinomas	32 (62.75)	62 (49.21)	NS
*N* (%) of patients with			
Stage I	48 (94.12)	96 (76.19)	<0.01
Stage II	3 (5.88)	28 (22.22)	<0.01
Stage III	0	1 (0.79)	
Stage IV	0	1 (0.79)	

NS = not significant.

**Table 3:** The comparison of anthropometric and biochemical parameters and clinical stage in the PTC patients depending on the presence/absence of the metabolic syndrome.

	Patients without the metabolic syndrome *n* = 109 95 women, 14 men (mean ± SD; median)	Patients with the metabolic syndrome *n* = 68 56 women, 12 men (mean ± SD; median)	*p*
Age	47.21 ± 14.45; 46	57.06 ± 11.54; 59.5	<0.001
BMI (kg/m^2^)	26.19 ± 5.06; 26.15	31.11 ± 4.52; 31.06	<0.001
Waist (cm)	86.77 ± 13.75; 87	101.70 ± 11.96; 101	<0.001
WHR	0.83 ± 0.09; 0.82	0.91 ± 0.08; 0.90	<0.001
Glucose (mg/dL)	117.80 ± 22.30; 114	125.18 ± 29.22; 119	NS
TGs (mg/dL)	133.22 ± 67.16; 112	187.07 ± 61.44; 178	<0.001
HDL (mg/dL)	62.28 ± 18.32; 58	49.28 ± 16.65; 44.5	<0.001
Cholesterol (mg/dL)	281.22 ± 122.56; 231	271.04 ± 119.41; 230	NS
HOMA-IR	1.14 ± 0.65; 0.97	1.68 ± 1.25; 1.41	<0.001
Adiponectin (*μ*g/mL)	15.66 ± 6.44; 15.0	13.64 ± 6.05; 13.0	<0.001
Leptin (ng/mL)	14.14 ± 10.26; 10.98	26.08 ± 24.52; 21.5	<0.001
Chemerin (ng/mL)	213.22 ± 49.22; 209	243.75 ± 44.79; 236	<0.001
Vitamin D (nmol/L)	67.53 ± 24.05; 61	63.02 ± 21.25; 60	NS
IL-6 (pg/mL)	2.49 ± 0.76; 2.29	3.01 ± 1.28; 2.83	<0.001
*N* (%) of patients with microcarcinomas	62 (56.88)	32 (47.06)	NS
*N* (%) of patients with			
Stage I	94 (86.24)	50 (73.53)	<0.05
Stage II	14 (12.84)	17 (25)	<0.05
Stage III	1 (0.92)	0	
Stage IV	0	1 (1.47)	

NS = not significant.

**Table 4:** Comparison of anthropometric and biochemical parameters in pT1a patients (*n* = 94) and patients > pT1a (T1b, pT2, pT3, and pT4; *n* = 83).

	pT1a, *n* = 94 83 women, 11 men (mean ± SD; median)	>pT1a, *n* = 83 68 women, 15 men (mean ± SD; median)	*p*
Age	49.73 ± 14.35; 53	52.42 ± 13.99; 56	NS
BMI (kg/m^2^)	27.99 ± 6.16; 27.22	28.18 ± 4.44; 28.33	NS
Waist (cm)	91.50 ± 16.18; 90.5	93.55 ± 13.42; 95.5	NS
WHR	0.85 ± 0.09; 0.85	0.87 ± 0.09; 0.88	NS
Glucose (mg/dL)	122.26 ± 29.73; 117	1180.98 ± 19.54; 116	NS
TGs (mg/dL)	153.23 ± 70.25; 144.5	163.80 ± 69.31; 161.5	NS
HDL (mg/dL)	58.30 ± 18.73; 54	53.85 ± 18.46; 52	NS
Cholesterol (mg/dL)	273.46 ± 118.22; 231	279.64 ± 124.25; 230	NS
HOMA-IR	1.37 ± 1.12; 1.07	1.29 ± 0.69; 1.14	NS
Adiponectin (*μ*g/mL)	15.45 ± 7.24; 13.5	14.38 ± 5.17; 14.0	NS
Leptin (ng/mL)	18.68 ± 19.18; 14.77	17.92 ± 15.66; 13.5	NS
Chemerin (ng/mL)	221.90 ± 48.22; 221	226.48 ± 51.76; 215.5	NS
Vitamin D (nmol/L)	66.92 ± 25.59; 61	64.82 ± 19.93; 63	NS
IL-6 (pg/mL)	2.69 ± 0.97; 2.37	2.66 ± 1.04; 2.5	NS
Metabolic syndrome *n* (%)	32 (34.04)	36 (43.37)	NS

NS = not significant.

**Table 5:** Relations of anthropometrical and biochemical factors to clinical stage, applied in logistic regression, according to AJCC/TNM 8th edition, before and after adjustment for sex.

	OR (95% CI)	*p*
BMI (≥25 vs. <25)	5.0 (1.44; 17.36)	<0.05
BMI/sex	4.78 (1.35; 16.93)	<0.05
Waist circumferences (≥88 cm vs. <88 cm in women, ≥102 cm vs. <102 cm in men)	6.23 (1.78; 21.89)	<0.01
Waist circumferences/sex	6.35 (1.78; 22.69)	<0.01
HOMA-I (≥2.0 vs. <2.0)	2.2 (0.75; 6.66)	NS
HOMA-I/sex	2.18 (0.71; 6.71)	NS
Leptin (high tertile vs. low tertile)	3.9 (1.18; 13.01)	<0.05
Leptin/sex	4.8 (1.35; 17.01)	<0.05
Adiponectin (low tertile vs. high tertile)	0.94 (0.36; 2.46)	NS
Adiponectin/sex	0.75 0.25; 2.19	
Chemerin (high tertile vs. low tertile)	2.9 (1.08; 7.78)	NS
Chemerin/sex	2.9 (1.08; 7.75)	NS
IL-6 (high tertile vs. low tertile)	8.05 (2.19; 29.56)	<0.01
IL6/sex	7.42 (1.99; 27.54)	<0.001
Vitamin D (high tertile vs. low tertile)	0.82 (0.29; 2.29)	NS
Vitamin D/sex	0.83 (0.30; 2.33)	NS

NS = not significant.

**Table 6:** Spearman correlations between HOMA-I and anthropometric and biochemical characteristics in all the PTC patients.

Parameter	*R* Spearman	*p*
BMI	0.32	<0.001
Waist circumference	0.27	<0.001
WHR	0.18	<0.05
TGs	0.27	<0.01
HDL	−0.21	<0.05
Cholesterol	−0.06	NS
Leptin	0.34	<0.001
Adiponectin	−0.21	<0.01
Chemerin	0.20	<0.05
Vitamin D	−0.06	NS
IL-6	0.20	<0.05

## Data Availability

The data used to support the findings of this study are restricted by the Bioethics Committee of the Medical University of Silesia and by the Bioethics Committee of the Maria Sklodowska-Curie Memorial Cancer Centre and Institute of Oncology, Gliwice Branch, Poland, in order to protect patient privacy. Data are available from the corresponding author (Lucyna Siemińska, MD) for researchers who meet the criteria for access to confidential data.
